# IMproving PArticipation of patients in Clinical Trials - rationale and design of IMPACT

**DOI:** 10.1186/1471-2288-10-85

**Published:** 2010-09-27

**Authors:** Katrien Oude Rengerink, Brent C Opmeer, Sabine LM Logtenberg, Lotty Hooft, Kitty WM Bloemenkamp, Monique C Haak, Martijn A Oudijk, Marc E Spaanderman, Johannes J Duvekot, Christine Willekes, Maria G van Pampus, Martina M Porath, Jim van Eyck, Marko J Sikkema, Ben Willem J Mol

**Affiliations:** 1 Department of Obstetrics and Gynaecology, Academic Medical Centre, Amsterdam, The Netherlands; 2 Department of Clinical Epidemiology, Biostatistics and Bioinformatics, Academic Medical Centre, Amsterdam, The Netherlands; 3 Department of Obstetrics and Gynaecology, Onze Lieve Vrouwe Gasthuis, Amsterdam, The Netherlands; 4 Dutch Cochrane Centre, Academic Medical Centre, Amsterdam, The Netherlands; 5 Department of Obstetrics, Leiden University Medical Center, Leiden, The Netherlands; 6 Department of Obstetrics and Gynaecology, VU Medical Center, Amsterdam, The Netherlands; 7 Department of Obstetrics and Gynaecology, University Medical Center Utrecht, Utrecht, The Netherlands; 8 Department of Obstetrics and Gynaecology, Radboud University Medical Center, Nijmegen, The Netherlands; 9 Department of Obstetrics and Gynaecology, Erasmus University Medical Center, Rotterdam, The Netherlands; 10 Department of Obstetrics and Gynaecology, Maastricht University Medical Center, Maastricht, The Netherlands; 11 Department of Obstetrics and Gynaecology, University Medical Center Groninngen, The Netherlands; 12 Department of Obstetrics and Gynaecology, Maxima Medical Center, Veldhoven, The Netherlands; 13 Department of Obstetrics and Gynaecology, Isala Clinics, Zwolle, The Netherlands; 14 Department of Perinatology and Gynaecology, Hospital Group Twente, Almelo, The Netherlands

## Abstract

**Background:**

One of the most commonly reported problems of randomised trials is that recruitment is usually slower than expected. Trials will cost more and take longer, thus delaying the use of the results in clinical practice, and incomplete samples imply decreased statistical power and usefulness of its results. We aim to identify barriers and facilitators for successful patient recruitment at the level of the patient, the doctor and the hospital organization as well as the organization and design of trials over a broad range of studies.

**Methods/design:**

We will perform two cohort studies and a case-control study in the Netherlands. The first cohort study will report on a series of multicenter trials performed in a nationwide network of clinical trials in obstetrics and gynaecology. A questionnaire will be sent to all clinicians recruiting for these trials to identify determinants - aggregated at centre level - for the recruitment rate. In a case control-study nested in this cohort we will interview patients who refused or consented participation to identify factors associated with patients' consent or refusal. In a second cohort study, we will study trials that were prospectively registered in the Netherlands Trial Register. Using a questionnaire survey we will assess whether issues on hospital organization, trial organization, planning and trial design were associated with successful recruitment, i.e. 80% of the predefined number of patients recruited within the planned time.

**Discussion:**

This study will provide insight in barriers and facilitators for successful patient recruitment in trials. The results will be used to provide recommendations and a checklist for individual trialists to identify potential pitfalls for recruitment and judge the feasibility prior to the start of the study. Identified barriers and motivators coupled to evidence-based interventions can improve recruitment of patients in clinical trials.

## Background

Evaluation research is essential to inform evidence based health care decisions. The randomized controlled trial (RCT) is worldwide considered as the best instrument to evaluate the effectiveness of medical interventions. One of the most commonly reported problems with the conduct of such RCTs, however, is that recruitment is usually slower than expected. In the 1970's an American pharmacologist, Luis Lasagna, stated that once trial recruitment starts, the supply of eligible patients becomes a fraction of what was assumed before the start of the trial. This phenomenon, currently known as Lasagna's Law, still holds today [1,2]. In the UK, in a cohort of 114 multicenter trials funded by the UK Medical Research Councel and the UK Health Technology Assessment Programme between 1994 and 2002, less than one-third recruited their original target within the time originally planned, and around one third had extensions [1].

If in a clinical trial the targeted sample size is not achieved, it will have less statistical power to convincingly demonstrate potentially important differences between the groups, which might make the results less useful or not at all applicable in clinical practice - it will not improve practice and wastes the contribution of participants who already participated. In addition, if recruitment has to be extended to reach the required sample size, the trial will cost more and take longer, thus delaying the use of its results in clinical practice. As the total amount of funding is limited, fewer trials can be conducted and hence less clinical dilemmas can be solved.

**Figure 1:**
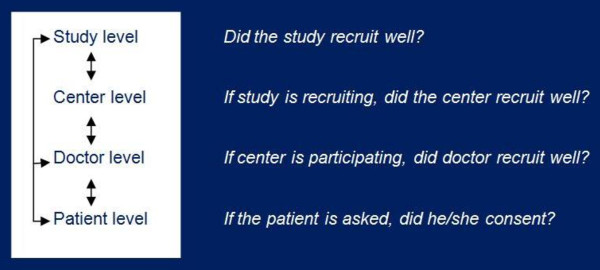
**Factors for lack of recruitment can be found on different levels: with interactions between levels**.

Reasons for lack of recruitment can be found at different levels: the patient, the doctor, the participating centre or department, the study organisation and the study design (see figure 1) [1,3-7].

Rendell et al. reviewed incentives and disincentives to participation, focussing on participation of clinicians [6]. Lovato et al. and Ross et al. reviewed barriers to both patient and clinician participation [4,8]. Other reviews were dedicated to a specific disease or group of diseases [3,9-13]. Frequently mentioned barriers for patients are preference for one form of treatment, concerns with the trial setting, dislike of randomisation, general discomfort with the research process, distrust in researchers, complexity and stringency of the protocol, presence of a placebo or no-treatment group, potential side effect, fear that trial involvement would have a negative effect on the relationship with their physician and their physicians attitudes towards the trial, the potential for increased demands and the mere inability to make a decision [13,14]. Frequently mentioned barriers to clinician participation are time constraints, lack of staff and training, worry about the impact on doctor-patient relationship, concern for patients, loss of professional autonomy, difficulty with the consent procedure, lack of rewards and recognition, and an insufficiently interesting question [4].

These reasons might be different across specialties, countries or due to the nature of the disease or disease population, but will probably also have common denominators [10]. In many of the included papers in the reviews it is uncertain if and how these results can be generalized to other trials and populations. In the reviews there might also be an over representation of very successful or very unsuccessful recruiting trials, as especially these trials might invoke a study of determinants of recruitment by the trial coordinator. Recently, the STEPS study focused on recruitment from different perspectives, and although they identified determinants, they did not have sufficiently definitive results to make strong recommendations [1].

In conclusion, although several potential barriers have been identified, it remains unclear whether they are applicable for any next trial. At present, neither a rule which can predict successful recruitment, nor a checklist to assess the feasibility of recruitment prior to the start of a trial, or even prior to a funding decision, is available. We therefore aim to study methods for IMproving PArticipation in Clinical Trials (IMPACT). We aim to identify predictors for (un)successful patient recruitment at the level of the patient, the doctor and the study and organizational level in order to provide a rule or checklist for successful patient recruitment in clinical trials.

## Methods/design

To address this topic at different relevant levels, we plan two cohort studies and a nested case control study in The Netherlands. In the first cohort study we will focus on successful recruitment at the level of participating centres, where characteristics of the clinician will be aggregated at the level of the centre. In a case control study embedded in this cohort study we will focus on the patient level. In the second cohort study we will identify determinants for success of recruitment in a cohort of trials registered in the national Netherlands Trial Register.

This study did not require formal approval of an ethics committee or internal review board, as was confirmed by the ethics committee of the Academic Medical Center. We interpreted completion and return of a questionnaire as the respondent's consent for participation. Methodological details of these studies are described below.

### Cohort study 1: predicting recruitment at centre level

The first cohort aims to determine which factors on centre level will influence recruitment of patients. The cohort will consist of a series of multicenter trials performed in a nationwide Consortium on studies in Obstetrics and Gynaecology, in which currently over 70 medical centres participate (table 1) [15]. At the centre level, we will study aspects of the doctor as well as aspects of the organisation. A questionnaire will be sent to gynaecologists, residents, research nurses, and midwives in the Netherlands who work in centres recruiting for these trials. We will also collect data on characteristics of clinicians and study organization.

**Table 1:** A cohort of studies performed in a nationwide Consortium on studies in obstetrics and gynaecology in the Netherlands.

Nr	Trial	Population	Sample size
**1**	Amphia	Women with a multiple pregnancy before 20 weeks pregnancy	720

**2**	Hypitat II	Women with pregnancy induced hypertension or mild pre-eclampia at 34-37 weeks gestation	400

**3**	Digitat	Women with a singleton pregnancy at 36 completed weeks of gestation or more	626

**4**	Ppromexil	Women with preterm prelabour rupture of membranes between 34 and 37 weeks gestation	520

**5**	Stan	Women in labour over 36 weeks of gestation with an indication for CTG monitoring	2400

**6**	Apostel 1	Women with threatened preterm labour at 24-34 weeks gestation.	220

**7**	Apostel 2	Women with threatened preterm labour at 26-32+2 weeks gestation.	400

**8**	TRIPLE P	Women with a singleton pregnancy	1920

**9**	Probaat	Women ≥ 37 weeks of gestation and Bishop score < 6	812

**10**	PreCare	Women with preeclampia or HELLP in previous pregnancy	250

**11**	WOMB	Women with >1000 mL postpartum fluxus	400

**12**	Truffle	Women at 26-32 weeks gestation with a fetus with intrauterine growth retardation.	500

**13**	Allo	Women with suspected fetal asphyxia during labour	220

**14**	ProTWIN	Women with a multiple pregnancy between 12 and 20 weeks gestation	660

**15**	Metex	Women with an extra uterine gravidity	72

**16**	ESEP	Women with an extra uterine gravidity in one of the tubae and a normal contralateral tuba	450

**17**	INeS	Couples with unexplained subfertility or a mild male factor	600

**19**	MOVIN'	Women with anovulation not pregnant after 6 ovulatory cycles of clomid	200

**20**	Bedrest	Women who undergo intra-uterin insemination	250

**21**	VUSIS 1	Women with stress-incontinence	100

**22**	PORTRET	Women with stress incontinence aged 35-80 years	400

**23**	CUPIDO	Women with vaginal prolaps	114

**24**	Pompoen	Women with postmenopausal bleeding	200

Based on these data, we will construct a prediction model. The primary outcome will be the percentage of eligible patients recruited, defined as the number of randomized patients per centre divided by the number of eligible patients. Secondary outcomes will be the number of randomized patients divided by the number of available patients per centre stratified per study. The number of eligible patients is registered for a part of the studies and will be estimated based on the LVR, the Dutch national perinatal registry.

As potential predictors we will take into account characteristics of the clinicians, e.g. the proportion of doctors with a PhD, as well as clinicians' views on the trial, e.g. prior belief in the relevance and quality of the study design. We will also collect characteristics of the study organisation: e.g. status of the hospital (academic, teaching or general), availability of research nurses or employees for counselling of the studies, clarity of research protocol and logistics, responsibilities for recruitment. These potential predictors reflect clinicians' views on these topics, aggregated at the level of department.

For reliable modelling on prediction experts recommend that there should be at least about 10 events in the data set for each potential predictor to be included in the model [16]. As we expect a response in about 65 of 70 centres, prediction models can include about 6-7 predictors at centre level.

Missing values will be imputed. The predictive accuracy of the model will be assessed using calibration, which evaluates the correspondence between the model's predicted percentage of randomized patients and the observed percentage of randomized patients. The discriminative ability of the prognostic model will be assessed by using receiver operating characteristics (ROC) analysis. We will then perform internal validation using bootstrapping and apply shrinkage to correct for over fit. A simplified prediction rule will be derived from the regression coefficients of the independent predictors in the multivariable model. If a valid prediction rule cannot be constructed, we will use the multivariable model to identify risk factors for a low recruitment percentage.

### Case-control study: predicting recruitment at patient level

In a case control-study nested in this cohort we will interview patients who refused or consented participation in a set of clinical trials, to identify factors which influenced the decision to participate.

We will perform qualitative semi-structured interviews. The interviews will start with open questions on the motivation of a patient to consent or refuse participation. Subsequently, the interview will be guided by a topic list that is based on the literature and on input from experienced trialists. Topics will include counselling, clarity and understanding of patient information, knowledge of and attitude towards scientific research [9,17], attitude towards the doctor or health care organisation, type of intervention, practical considerations and organisational issues, (perceived) personal benefit, (dis)trust and their social demographic characteristics. This topic list will be tailored during the study.

We will interview patients who were recently counselled for participation in a RCT in a broad range of trials in the field of obstetrics, subfertility, gynaecology, internal medicine, neurology and surgery. Patients will be selected using purposive sampling. We will interview patients from different doctors, different natures of disease, different levels of education, and different regions in the Netherlands. The number of patients to be interviewed will be dependent on the variety of responses until saturation of the data is reached. We expect this to be about 10 patients who participated and 10 patients who refused for the first subspecialty obstetrics. As we assume that reasons for the decision about participation share common components across specialties, we can complete the reasons with a smaller sample of patients from other clinical specialties. This will provide us information about the full spectrum of barriers and facilitators for participation.

Based on these barriers and motivators observed in the interviews we will construct a questionnaire to quantify these findings in a representative sample of patients in the fields of obstetrics and gynaecology, neurology, surgery and internal medicine.

### Cohort study 2: Predicting recruitment success of trials registered in the Netherlands Trial Register

In a second cohort study, we will include study trials that have been registered prospectively in the Netherlands Trial Register. The cohort of studies will consist of all studies that registered their stop date between January 1^st ^2005 and January 1^st^, 2010 (expected number of available trials N≈1000). Using a questionnaire survey, we will investigate whether issues on hospital organization, trial organization, planning and trial design are predictive for successful recruitment, defined as ≥80% of the patients recruited within the time frame defined in the grant application.

As potential predictors we will take into account trial characteristics, i.e. placebo arm, blinding, experimental status of the intervention to be evaluated; characteristics of the trial organisation, i.e. research staff available to counsel patients and acquire follow up data, who is responsible for recruitment; and characteristics of the principal investigator and the research group, i.e. composition of different expertise, experience and training in trial research. For reliable prediction modelling experts recommend that there should be at least about 10 events in the data set for each potential predictor to be included in the model [16]. In a sample of 1000 studies, with a (low) recruitment rate of 30% about 30 potential predictors can be tested reliably.

Missing values will be imputed. Like in the cohort study I, the predictive accuracy of the model will be assessed using calibration, which evaluates the correspondence between the model's predicted probabilities of recruitment success and the observed recruitment success over groups. The discriminative ability of the prognostic model will be assessed by using receiver operating characteristics (ROC) analysis.

We will perform internal validation using bootstrapping and apply shrinkage to correct for over fit.

A prediction rule will be derived from the regression coefficients of the independent predictors in the multivariable prognostic model. If a valid prediction rule cannot be constructed, we will use the model to identify risk factors for a low recruitment percentage.

## Discussion

At the end of the study, we will have an inventory of predictors, barriers and facilitators for successful patient recruitment in trials. The first cohort study will provide information on prediction of recruitment in different centres participating in obstetrical, subfertility and gynaecological trials. From the nested case-control study we will obtain qualitative and quantitative information about factors which influence patient participation. The second cohort study will provide insight in factors related to successful recruitment at study level. Based on the prediction models or risk factors for unsuccessful recruitment, we will develop a set of recommendations and a checklist that can be used by individual trialists before the start of the study to assess if recruitment of the proposed sample size with their strategy will be feasible.

A strong point of the design is that we will address recruitment factors at different levels and from different perspectives, throughout a variety of trials in various fields of medicine. This will provide us with a broad overall picture of reasons why patients participate or refuse participation, and will provide insight in a common denominator between trials, or clarify differences. At the same time this is a potential pitfall: the reasons for participation or non-participation in a clinical trial might predominantly depend on exclusive characteristics of a trial and its targeted population, so that general predictors may not be identified. If so, the results are still valuable, but we should focus on the development of a general applicable recruitment tool. Such a tool might consist of a strategy based on interviewing a number of eligible participants as well as a number of recruiters and/or clinicians prior to start or during the piloting of the trial.

Another strong point is that the first cohort is based on clinicians recruiting for a set of trials from the nationwide consortium in Obstetrics and Gynaecology. All academic medical centres and the majority of the Dutch hospitals recruit for trials running in this consortium, which enables us to cover a large part of the Netherlands without selection of the explicitly research minded hospitals. Moreover, the second cohort of studies from the Dutch Trial Register will be a representative sample of all trials performed in the Netherlands, since from July 2005 registration is required for publication in important journals. It will be a challenge to deliberately handle the heterogeneity of the trials included.

There are also some limitations in this study which require a remark. First, although we focus on different levels (level of the patient, centre and study) it is not possible to directly link the data from these levels. We therefore cannot disentangle the relative impact of each level to the recruitment problems. However, we will be able to provide a satisfactory estimation, given the variability between different specialties and trials.

Second, as we focus on trials in obstetrics and gynaecology in the first cohort study, generalisability of these results to other clinical specialties should be evaluated. As in this study it is not feasible to survey all specialties in depth, we think it is more informative to have a complete picture of one specialty over a limited amount of information from many specialties.

Furthermore, in the second cohort on predicting successful recruitment on study level, the predefined recruitment target will probably - as most power calculations - be based on limited information. When information emerges from external sources or interim analyses, the sample size might be adapted, which can make the trial more successful or efficient without reaching the originally planned sample size. However, sample size targets are taken into account when funding decisions are made. The extent to which a trial meets initial expectations can be viewed as a legitimate marker of trial success. Moreover, recruitment can be viewed of as a surrogate marker of more significant markers of success, such as the extent to which the trial question has been successfully addressed [1].

Interviewing patients from different specialties and trials will provide a broad spectrum of why patients participate or refuse. This will limit the number of patients interviewed from one trial, but the quantification of these reasons in a representative group of patients will allow us to examine differences between these specialties and trials.

Finally, as this study will be performed in the Netherlands, the Dutch health care system as well as the position of medical research in the Netherlands might influence its results. Existing literature can be used to compare our results with those from other countries. We realize that especially to make this study a success we need a high response rate to avoid selection of highly motivated researchers, patients and clinicians.

In conclusion, this design allows us to identify determinants for unsuccessful recruitment. Identified predictors for unsuccessful recruitment can be coupled to evidence-based strategies to improve recruitment in trials [7].

## Competing interests

The authors declare that they have no competing interests.

## Authors' contributions

KOR was involved in the design of the study and in drafting the manuscript, BCO was involved in the design of the study and critically revised the manuscript, SL critically revised the manuscript, LH was involved in the design of the study and critically revised the manuscript, KWM critically revised the manuscript, MCH critically revised the manuscript, MAO critically revised the manuscript, MES critically revised the manuscript, JJD critically revised the manuscript, CW critically revised the manuscript, MGvP critically revised the manuscript, MMP critically revised the manuscript, JvE critically revised the manuscript, MJS critically revised the manuscript, BWJM was involved in the design of the study, drafting the manuscript and critically revised the manuscript.

All authors have given final approval of the version to be published.

## Pre-publication history

The pre-publication history for this paper can be accessed here:

http://www.biomedcentral.com/1471-2288/10/85/prepub
